# MetaFIND: A feature analysis tool for metabolomics data

**DOI:** 10.1186/1471-2105-9-470

**Published:** 2008-11-05

**Authors:** Kenneth Bryan, Lorraine Brennan, Pádraig Cunningham

**Affiliations:** 1 Complex & Adaptive Systems Laboratory (CASL), University College Dublin, Ireland; 2 UCD School of Agriculture, Food Science & Veterinary Medicine, UCD Conway Institute, University College Dublin, Ireland

## Abstract

**Background:**

Metabolomics, or metabonomics, refers to the quantitative analysis of all metabolites present within a biological sample and is generally carried out using NMR spectroscopy or Mass Spectrometry. Such analysis produces a set of peaks, or *features*, indicative of the metabolic composition of the sample and may be used as a basis for sample classification. Feature selection may be employed to improve classification accuracy or aid model explanation by establishing a subset of class discriminating features. Factors such as experimental noise, choice of technique and threshold selection may adversely affect the set of selected features retrieved. Furthermore, the high dimensionality and multi-collinearity inherent within metabolomics data may exacerbate discrepancies between the set of features retrieved and those required to provide a complete explanation of metabolite signatures. Given these issues, the latter in particular, we present the MetaFIND application for 'post-feature selection' correlation analysis of metabolomics data.

**Results:**

In our evaluation we show how MetaFIND may be used to elucidate metabolite signatures from the set of features selected by diverse techniques over two metabolomics datasets. Importantly, we also show how MetaFIND may augment standard feature selection and aid the discovery of additional significant features, including those which represent novel class discriminating metabolites. MetaFIND also supports the discovery of higher level metabolite correlations.

**Conclusion:**

Standard feature selection techniques may fail to capture the full set of relevant features in the case of high dimensional, multi-collinear metabolomics data. We show that the MetaFIND 'post-feature selection' analysis tool may aid metabolite signature elucidation, feature discovery and inference of metabolic correlations.

## Background

Metabolites are the small molecular intermediates and products of an organism's metabolism. The set of metabolites present within an organism form its 'metabolome' [1]. The comprehensive and quantitative analysis of the whole metabolome is referred to as 'metabolomics' (or metabonomics) [2]. The most commonly used experimental techniques for measuring the global metabolome are Nuclear Magnetic Resonance Spectroscopy (NMR) and Mass Spectrometry (MS). Both experimental methods produce spectral profiles which can be indicative of the metabolic composition of experimental samples. After various pre-processing steps, a set of 'binned' spectral regions (or peaks) is produced for each sample [3]. These spectral regions or peaks may be viewed as a set of 'features' that may be used to characterize and discriminate between sample classes i.e. disease states, drug effects and cell types.

Using the set of features produced by the above techniques, several forms of data analysis may be performed depending on the aim of an investigation [4,5]. If sample class labels are unavailable, or in cases where the presence of novel classes is suspected, 'unsupervised' classification may be used to discover sample groupings. Data transformation methods such as Principal Component Analysis (PCA) may be sufficient to reveal class structure within the samples [6]. Hierarchical and partitional cluster analysis may also be applied to model sample relationships, within either the transformed or original feature space [7,8]. When class labels are available they may be used to support 'supervised' classification. Predictive models or 'classifiers' may then be built to classify unlabelled data. Linear Discriminant Analysis (LDA) has been applied to build a classification model from metabolomics data [9]. However due to the multi-collinear nature of metabolomics data it is better practice to perform Principal Component Analysis (PCA) prior to the application of LDA (PCA-LDA) [10]. Discriminant Analysis based upon Partial Least Squares (PLS-DA) has also become popular within the metabolomics domain [11]. PLS-DA uncovers the latent variables within the data that both model the feature values and separate the sample classes. Recently this technique has been enhanced in the form of Orthogonal-PLS-DA (O-PLS-DA) [12,13]. Other supervised methods used in this domain include Support Vector Machines (SVMs) and Artificial Neural Networks (ANN) [14-16].

A topic that overlaps somewhat with supervised classification is that of supervised feature selection. Feature selection may be employed to improve a classification model, in terms of generalization performance and accuracy, by eliminating non-informative features. Aside from this, feature selection may also be used to gain further insight into the rationale underlying class divisions within a particular domain. In the context of metabolomics, retrieving the set of class discriminating features may aid in the identification of the class determining metabolites. This may allow further elucidation of the system (e.g. disease mechanism) under investigation.

**Figure 1:**
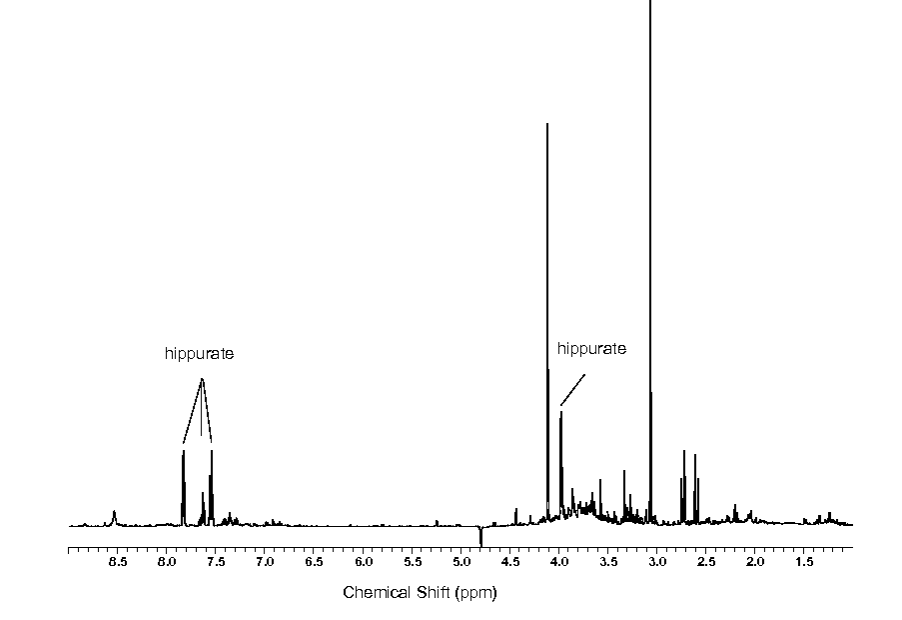
**The NMR spectrum of a urine sample**. A typical NMR spectrum of a urine sample [25]. Peaks due to the metabolite hippurate are highlighted.

However, features selected on the basis of classification accuracy, i.e. features that are sufficient to separate classes, may not always translate directly into an explanation that makes sense from the perspective of the bio-analytical scientist. This is often the case in NMR metabolomics data, where a metabolite may be represented by one or more spectral features. In this case a subset of these features (part of the metabolite signature) may provide a perfect classification model. For example, the peaks 3.97 ppm, 7.55 ppm, 7.57 ppm and 7.85 ppm, which represent hippurate, can be seen in Figure 1. As these peaks belong to the same metabolite they will vary together, or correlate, over a set of samples. From the perspective of sample classification such correlated peaks may provide redundant information. As a result, employing standard feature selection alone may risk omitting features that are important for metabolite signature identification. High dimensional NMR metabolomics data is replete with feature correlations (multi-collinearity) both within the signal (features relevant to class explanation) and noise (irrelevant features).

Feature selection methods less prone to the bias effects of multi-collinear data include those based on *variable influence on the projection *(VIP) values, derived from PLS-DA, and *variable importance *produced by a Random Forest ensemble classifier. Interestingly, there is a fundamental difference between Random Forest and PLS-DA. Unlike PLS-DA, Random Forest is a non-parametric technique and is unaffected by feature scale. For this reason, these techniques may be seen to be somewhat complementary. In both techniques other factors, such as noise within the dataset or threshold selection, may cause relevant features to be omitted from a selected set. As a result, some features important for classification explanation and metabolite signature identification may be not be retrieved.

**Figure 2:**
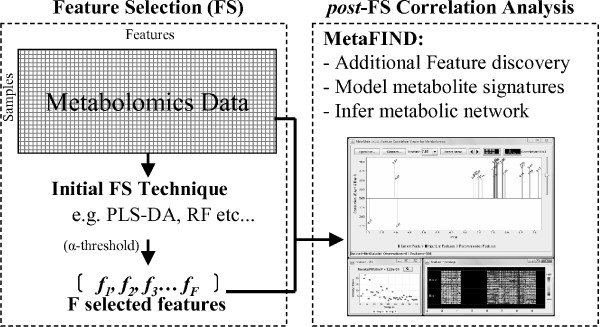
**The feature analysis pipeline**. The Metabolomics feature analysis pipeline incorporating MetaFIND as an additional post-feature selection step.

To further aid the retrieval of all peaks and metabolites relevant to both class discrimination and subsequent explanation, a novel metabolomics feature analysis tool called *MetaFIND *(*Meta*bolomics *F*eature *IN *terrogation and *D*iscovery) has been developed. The MetaFIND application addresses the multi-collinear aspect of metabolomics data by providing an adjunct to standard feature selection techniques. This takes the form of a 'post-feature selection' correlation analysis step, as illustrated in Figure 2.

The initial function of MetaFIND is to analyse the set of features retrieved by the investigator's chosen feature selection technique and provide support, via an interactive graphical interface, for uncovering the various metabolite signatures that may be present within this set. This provides a tool to bridge the gap between data driven class discrimination and domain explanation.

Secondly, MetaFIND also enables the user to examine correlations outside the selected feature set i.e. between the selected features and of the rest of the features in the dataset. This has the potential to discover novel features overlooked by the initial feature selection process. Any additional features retrieved may then further aid identification or discovery of metabolites. Lastly, MetaFIND allows the higher level correlations between metabolite signatures themselves to be examined. Both positively and negatively correlated metabolites signature may be uncovered. As discussed in [17], the identification of correlated metabolites may in turn contribute to the construction of the metabolic networks. Although some tools exist that enable analysis of spectral correlations [18,19], MetaFIND allows the user to dynamically examine the correlations of individual spectral features retrieved by arbitrary feature selection methods.

### Feature selection

In this study MetaFIND was applied, in conjunction with two alternative feature selection approaches, to investigate two metabolomics datasets. The first feature selection technique employed is referred to as Partial Least Squares (or lately Projection to Latent Structures) Discriminant Analysis (PLS-DA) and is one of the most commonly used techniques within metabolomics and chemometrics in general. In this study we also utilize a non-parametric feature selection method based on Random Forest classification. We now provide a brief discussion of both PLS-DA and Random Forest based feature selection strategies.

#### Partial Least Squares Discriminant Analysis

PLS Discriminant Analysis (PLS-DA) is a feature selection technique based upon the Partial Least Squares regression method for constructing predictive models developed by Herman Wold [20]. PLS is an improvement on the use of Multiple Linear Regression (MLR) in this context due to its ability to handle data in which features far out-number samples and in which many features are correlated (multi-collinearity). In PLS-DA the response variable is produced by encoding class labels as a binary vector of 1's and 0's indicating class membership. PLS extracts the set of *latent variables *which model the data but which are also correlated to the class membership vector. Once a PLS model has been built the influence of individual features may be captured with VIP (variable influence on the projection) measures derived from the PLS coefficients for the optimal set of components [11]. Features may then be ranked by VIP scores and selected after the choice of an appropriate threshold (usually *α *≥ 1). Clearly the chosen threshold value may greatly affect the set of retrieved features. PLS-DA is also scale dependent with the choice of scaling factor again affecting features selected [21]. Unit scaling (1/*SD*) can produce loadings that are difficult to interpret and result in artefacts and up-weighting of spectral noise. Mean centring alone favours features with higher intensity and variance. Even Pareto scaling (1/$\sqrt{SD}$) may enhance the contribution of metabolites present at high concentrations [22]. In this study we have applied this latter method of scaling to our features as a pre-processing step prior to PLS-DA.

#### Random Forest feature selection

Random Forest Classification was first proposed by Breiman [23]. This technique is based on growing many classification trees. A classification tree is an example of a supervised classification method in which feature values are used to build a model that enables the classification of unlabelled samples.

Random Forests may also be used as a basis to gain further insights into the data. One such extension allows Random Forest to assign *importance *values to features in terms of their influence on the classification accuracy of the forest and thus has been used to aid feature selection.

The influence of a particular feature on the classification accuracy of the Random Forest is referred to as its *importance *and may be evaluated by randomly permuting the feature over samples in each tree's 'out-of-bag' test set. These samples are then reclassified using the Random Forest. The difference in the number of correct classifications between the initial 'out-of-bag' data and the permuted 'out-of-bag' data is then divided by the number of trees in the forest and yields the importance value for that feature. The advantage of the Random Forest importance measure in feature selection over univariate screening methods is that it covers the impact of each feature individually as well as its multivariate interactions with other features. For example, Lunetta et al. find that genetic markers relevant in interactions with other markers or environmental variables can be detected more efficiently by means of Random Forests than by means of univariate screening methods like Fisher's exact test. As Random Forests are based on decision trees they also deal well with differently scaled features [24]. This is quite relevant to metabolomics data where the peaks may vary greatly in height (intensity).

#### Post-feature selection analysis

Given the high number of features and the high degree of feature collinearity in metabolomics data there is always potential, regardless of the feature selection technique employed, for the omission of explanatory features. The presence of noisy features, especially at lower scales (intensities) also adds to the risk of over-fitting and subsequent retrieval of irrelevant features. In the case of PLS-DA the investigator may use their experience to select an appropriate scaling technique to minimize the former risk while maximising the latter. Nevertheless the risk of omitting relevant features, especially of those present at low intensities, cannot be eliminated. The scale independent Random Forest based feature selection offers no such opportunity for feature weighting. As a result there is added risk of promoting low intensity noisy features which may demote relevant features that would aid explanation. In both approaches a balance between true and false positive rates must again be struck during the threshold selection step in which *F *features are chosen from the ranked list. Again the high dimensional, multi-collinear datasets found in metabolomics create further difficulties at this stage.

To help address these concerns it may be useful to carry out a second sweep of the dataset for additional features that may be relevant to the class explanation. One of the principal functions of MetaFIND is to conduct such a 'post-feature selection' analysis step. The implementation of this as well as MetaFIND's other functions will now be discussed in detail.

## Implementation

The MetaFIND application contains several components which support the user in the: (i) reconstruction of the class discriminating metabolite signatures, (ii) identification of additional relevant features omitted from the feature selection, (iii) identification of correlated metabolites which may aid the inference of the metabolic correlations at play in the system under investigation.

### The correlation graph

**Figure 3:**
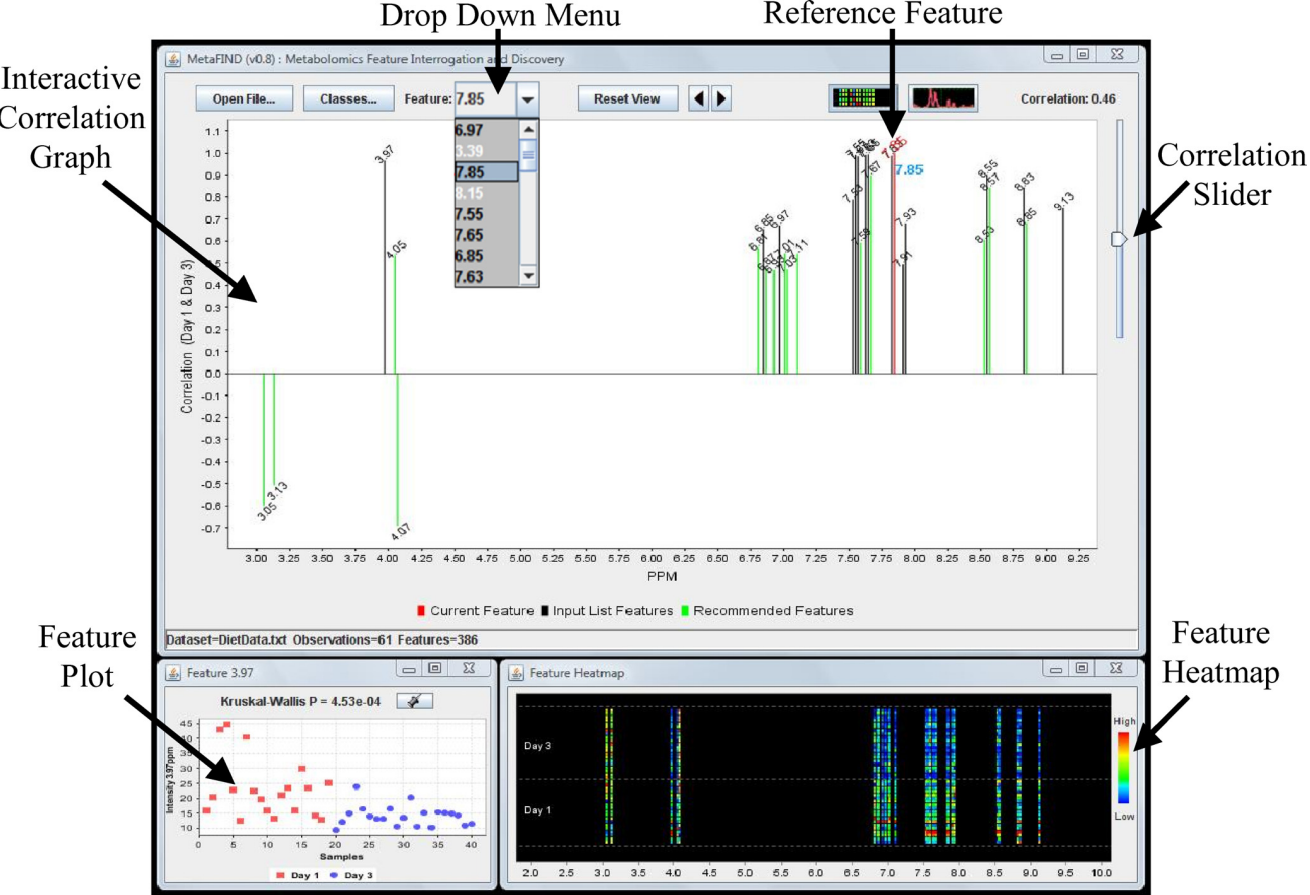
**MetaFIND screen shot**. The MetaFIND Application. Upon selection of the reference feature from the *Drop Down Menu *(features yet to be examined are displayed in white font) the Correlation Graph displays positively and negatively correlated features from the imported list (black). Novel *Recommended Features *are also displayed (green). Feature values may be examined globally (*Feature Heatmap*) and for selected features (*Feature Plot*). In this example the features most highly correlated (*r *≥ 0.92) to the *Reference Feature *represent the metabolite hippurate.

Central to the MetaFIND application is the *correlation graph *which provides a medium through which feature collinearity may be represented and analysed. In this graph the *y*-axis represents correlation. As this is derived from Pearson's correlation, it may render both positive (above the *x*-axis) and negative (below the *x*-axis) correlations. The *x*-axis represents the binned regions or peak names assigned to a feature. See Figure 3 for illustration.

The correlation graph enables the user to examine the intra-feature correlations between a feature retrieved by feature selection and the rest of features in the dataset. These features and the dataset are imported into MetaFIND. As mentioned in the introduction, the features, or peaks, which are representative of a particular class discriminating metabolite should be highly correlated over samples (having the same relative change in magnitude). Although this correlation may in some cases be reduced due to the cumulative effect of additional intensities in a region.

The user selects a *reference feature *to display from the imported list via a drop down menu above the correlation graph. Apart from displaying the list of imported features, this drop down menu directs the user to features yet to be examined within the selected set (white font). The currently displayed set of features (black font) and the previously viewed features (grey font) are also highlighted in this list. This function is particularly useful when a large set (e.g. 100 features) is imported. This function also means that a user may choose quite a liberal cut-off threshold during the initial feature selection stage.

Should the current reference feature represent a peak from a metabolite signature, the user may render this signature at a certain correlation threshold. Ordinarily the identification of this threshold and signature may prove a tedious pursuit with an investigator having to generate graphs at numerous correlation thresholds before happening upon the optimal rendering. However, MetaFIND extends this correlation graph concept into an interactive application in which thresholds may be tweaked and assessed in real-time (via the *correlation slider*). This allows a rapid retrieval of the optimal metabolite signature and potentially aids the identification of class discriminating metabolites.

The current reference feature is projected as a red line in the correlation graph. Correlated features above the chosen threshold appear as black lines (part of imported feature list) and green lines (new features not retrieved by feature selection) of appropriate height (correlation *y*) and position (spectral region *x*). Green lines represent *recommended features *and may represent part or all of a relevant metabolite signature overlooked by feature selection. If the reference feature represents part of a metabolite the rest of the features within this signature should appear at an optimal threshold. As the correlation is lowered below the optimal threshold more peaks will appear. These peaks may appear above and below the *x*-axis representing positive and negative correlations respectively. Correlated features may appear alone or in groups above the axis and may represent additional metabolite signatures which are positively correlated to the reference feature (and the metabolite it may partially represent). These signatures may represent additional, correlated metabolites, which increase and decrease in tandem with the reference feature (and metabolite) and may help infer metabolic correlations. Should these feature sets appear below the *x*-axis they may represent a negatively correlated metabolite signature. Again these features may be projected as either black or green lines depending on their presence or absence from the initial imported list.

The interactive correlation graph also contains an additional zoom function which is very useful when displaying a high number of features (e.g. 5 – 10,000). The user can then enlarge a region by simply clicking and dragging the cursor over the area of interest. Having selected the appropriate resolution the user may then scroll left or right using the arrow buttons above the correlation graph. Once a feature of interest is identified it may be further assessed by plotting its values over all samples. This is achieved by clicking on the projected feature which displays the *feature plot*.

### The feature plot

The feature plot facility supports the user in determining the relevance of a feature of interest by examining how its values change over samples. In this way feature magnitude (intensity), class discrimination and outlying samples may be quickly examined. The MetaFIND application also allows a feature plot to be retained on the desktop for reference and comparison with other features. In this way the user may also directly compare the feature vectors. This function is useful for determining the features which are representative of a single metabolite signature. The feature plot also contains a tool-tip function which allow feature identifiers to be displayed. Lastly, the feature plot also provides the *p*-value for each feature in the form of the non-parametric Kruskal-Wallis statistic. Although not demonstrated here, MetaFIND may be used to analyse feature significance over multiple sample classes. For simultaneous assessment of all currently displayed features the heatmap function may be also be used.

### The heatmap

The heatmap displays a colour representation of the values of all currently displayed features over all currently displayed samples. This function provides an alternative way to rapidly assess many features simultaneously. This display is also interactive returning feature names and values for individual samples. In practice the heatmap allows the user to rapidly assess the class separation and correlation of hundreds of features and direct the user to regions of interest. The user may then carry out a more detailed examination of these regions using the correlation graph and the feature plot functions. The heatmap is activated by clicking on the heatmap button above the correlation graph and it appears below the graph on the screen.

### The class spectrum

The class spectrum function in MetaFIND allows the user to display the mean class values for the currently displayed features. This class spectrum plot allows the user to view the mean magnitude of features over a sample class and aids preliminary identification of peaks. This again contains a tool-tip interaction and fulfils a role similar to that of the heatmap in directing the user to general regions of interest. The class spectrum is activated by clicking on the class spectrum button above the correlation graph. Once opened both the heatmap and the class spectrum displays are tied to the main correlation graph and dynamically change in response to the currently displayed features.

### Implementations

We used the R statistical computing language for the basic Random Forest implementation. This utilizes Breiman's original Fortran code for Random Forest and is also the most practical implementation available in having sufficient speed to handle the large features sets in Metabolomics Data. PLS-DA was carried out using SIMCA-P+ (Umetrics, Sweden). The MetaFIND feature analysis application is implemented in Java and utilizes the *JFreeChart *and *JCommons *packages. MetaFIND and related documentation are available at .

## Results and discussion

In this section we present an evaluation of the MetaFIND application using two metabolomics datasets described in section 3.1. The objective of this evaluation is to establish if MetaFIND can support the feature selection and subsequent metabolite identification processes. We employ MetaFIND in a post-feature selection capacity supporting diverse feature selection methods based on PLS-DA and Random Forests (see section 1.1).

### Metabolomics data

Two NMR datasets were used in the evaluation. The first dataset (Dataset 1) is derived from a previously published study [25]. This dataset consists of 60 spectral profiles (derived from urine samples of 21 subjects) over 387 spectral bins in the range 0.5–10 ppm. There are three classes of dietary intake corresponding to three different dietary regimes. The second dataset (Dataset 2) is derived from a NMR analysis of different brain region in rats [unpublished data]. It consists of 33 spectral profiles over 7901 spectral bins. The data contains 4 classes, pre-frontal cortex (PFC), hippocampus (H), cerebellum (C) and brain stem (BS). A sample of this dataset is available to download with the MataFIND application at . For further details, including experimental parameters and conditions, see [25].

### MetaFIND evaluation

#### Post-feature selection analysis using MetaFIND

**Figure 4:**
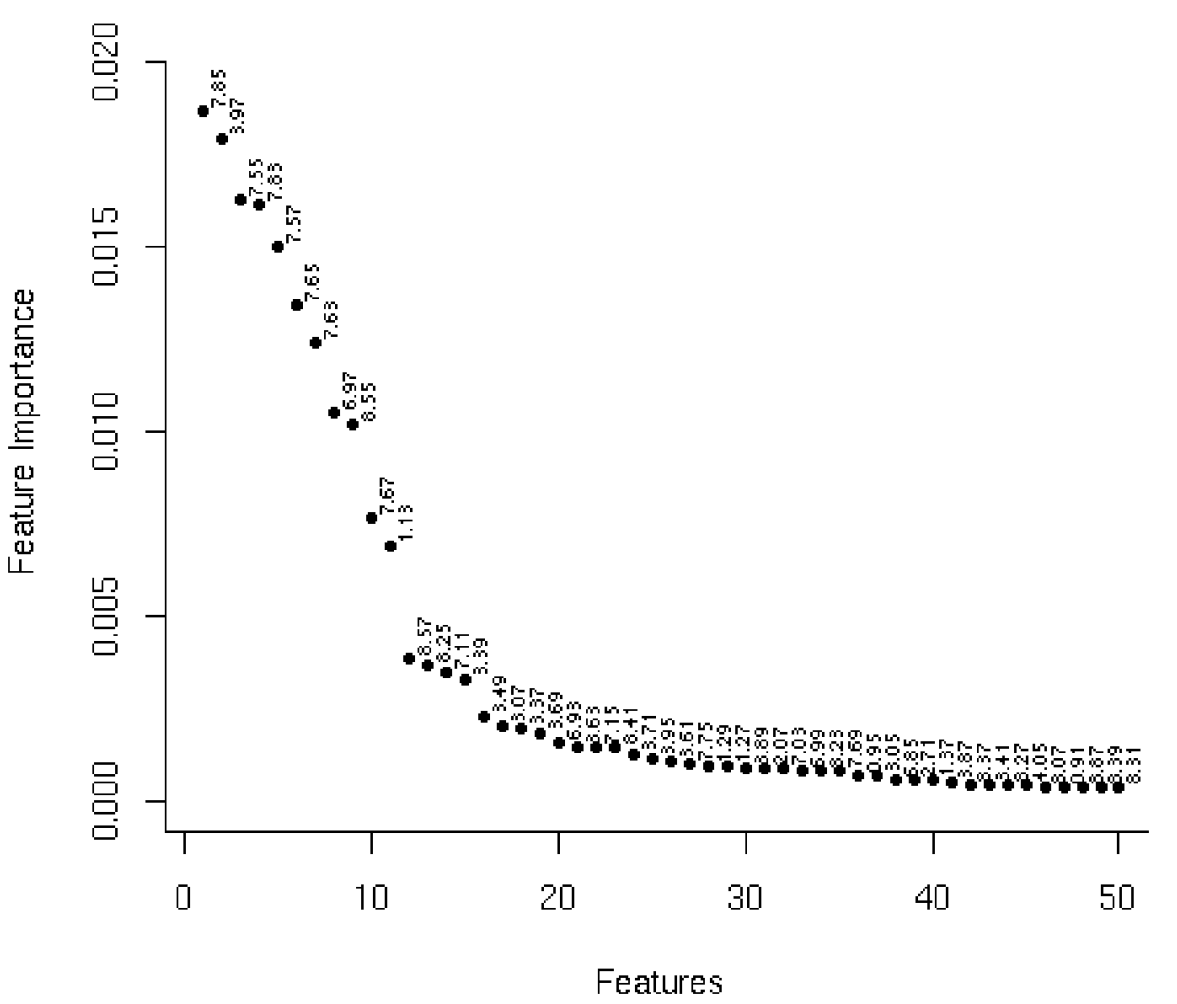
**Feature importance graph for the diet study (Dataset 1)**. Assessing the trend of importance scores may aid threshold selection in Random Forest feature selection. Presented here is the feature importance trend for Dataset 1.

In PLS-DA based feature selection using VIP scores a cut-off threshold of 1 was used to select the most important features. In the case of the Random Forest feature selection technique the variable importance plot was used to aid threshold selection, see Figure 4. In the diet dataset a sharp drop over the first 20 or so features is clearly visible in feature importance plot. In this diet data the top 20 features were selected for further analysis. A similar method was used to direct the selection of the top 100 features in the brain tissue dataset. Differing modes of threshold selection again create further diversity between the two feature selection methods used in this study. The aim of the remainder of this section is to evaluate how MetaFIND performs when used in conjunction with these alternative feature selection strategies.

### Dataset 1: diet study

**Figure 5:**
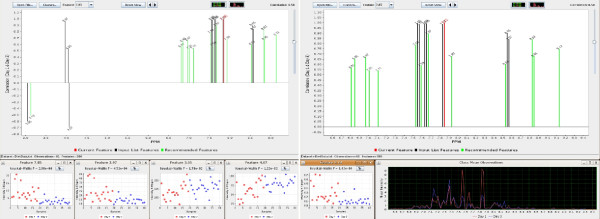
**Analysis of Dataset 1 using MetaFIND**. (a) Analysis of the reference feature 7.85 using MetaFIND. The features representing the hippurate signature (spectral regions 3.96–3.98 ppm, 7.54–7.56 ppm, 7.56–7.58 ppm, 7.84–7.86 ppm) are the first to appear above the *x*-axis.(b) Region 6.5–9.5 ppm enlarged. Features representing creatinine (spectral regions 3.05 ppm, 4.07 ppm) appear below the *x*-axis (anti-correlated). Feature 9.13 (nicotinic acid), omitted by PLS-DA feature selection, was uncovered using MetaFIND.

**Figure 6:**
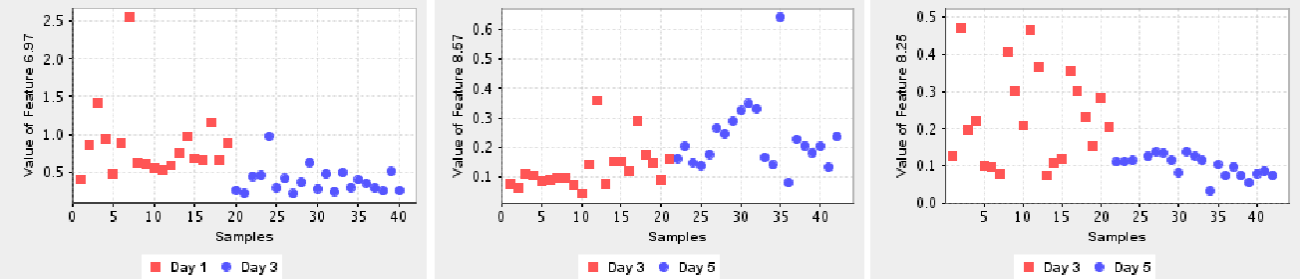
**Features discovered by MetaFIND from Dataset 1**. Feature plots for selected features that were retrieved by MetaFIND analysis, see Table 1 for the full set. The *y*-axis represents the intensity and the *x*-axis represents the samples sorted by class. Feature 9.13 was subsequently identified as the metabolite nicotinic acid.

The features selected by PLS-DA for Dataset 1 were imported into the MetaFIND application. Features selected as being discriminating between classes Day 1 and Day 3 were first examined. The metabolite signature modelling aspect of MetaFIND can be seen in Figure 5(a). Feature 7.85 (2.98 × 10^-6^) was selected as the reference feature and the correlation threshold was gradually lowered using the correlation slider. The first most highly correlated (*r *= 0.92) features to appear are those that represent hippurate (spectral regions 3.96–3.98 ppm, 7.54–7.56 ppm, 7.56–7.58 ppm, 7.84–7.86 ppm). As the correlation is further lowered the first features to appear below the *x*-axis are 3.05 and 4.07 which represent creatinine. Feature plots were then generated for 7.85 and 3.97 (bottom left) and showed these were present at a higher intensities on Day 1 compared to Day 3. Features representing creatinine (3.05, *p *= 1.78 × 10^-2 ^and 4.07, *p *= 1.22 × 10^-3^) are present at lower levels on Day 1 than on Day 3. Feature plots for these features are shown on the bottom right. The correlation plot suggest that the intensity of the features representing each metabolite may be anti-correlated. Several *recommended *features are also highlighted in green. In the region 6.5–9.5 ppm, as shown in Figure 5(b), the feature plot for the recommended feature 9.13 (*p *= 1.42 × 10^-6^) is displayed. Further investigation assigned this to nicotinic acid. This is an example of a feature overlooked during the initial PLS-DA feature selection that was representative of a metabolite that shows some class discrimination (*p *= 1.42 × 10^-6^), illustrating the potential benefit of the feature discovery aspect of MetaFIND. Apart from using the feature plot, recommended features may be assessed via the *class spectrum*, as shown bottom right in Figure 5(b). This can be used for a preliminary assessment of the feature class separation. The investigator may then return to the original spectral data for confirmation. Further features uncovered by MetaFIND are presented in Table 1 and Figure 6.

**Table 1:** MetaFIND uncovers additional features from Dataset 1.

**PLS-DA**								**Random Forest**							
**Day 1 vs Day 3**				**Day 3 vs Day 5**				**Day 1 vs Day 3**				**Day 3 vs Day 5**			
**FS**	**MF**	**P-value**	**VIP**	**FS**	**MF**	**P-value**	**VIP**	**FS**	**MF**	**P-value**	**Imp.Rank**	**FS**	**MF**	**P-value**	**Imp.Rank**
			
3.97	9.13*	1.42E-4	0.64	3.05	**8.55**	1.01E-5	0.61	6.97	3.97*	4.53E-4	26	7.85	3.05*	1.78E-2	36
7.85	**8.85**	9.99E-4	0.75	4.07	**8.57**	2.52E-4	0.53	3.39	7.57*	4.07E-5	21	3.97	4.07*	1.22E-3	50
7.55	**2.45**	5.62E-2	0.75	7.15	**8.25**	1.34E-3	0.35	7.85	4.05*	1.33E-3	30	7.55	3.07*	2.07E-4	22
7.83	**6.87**	5.06E-3	0.49	7.19				8.15	**8.57**	4.27E-3	57	7.83	**2.17**	5.57E-2	141
4.05	**8.05**	1.78E-2	0.65	3.31				7.55	**2.45**	5.62E-2	139	7.57	**7.75**	2.44E-3	30
7.57	**8.25**	4.09E-4	0.71	7.17				7.65				7.65	**6.93**	8.59E-4	23
7.65	**8.29**	7.64E-3	0.68	3.07				6.85				6.97			
7.63				3.13				7.63				7.63			
3.53				3.97				2.81				8.55			
8.55				7.85				7.83				7.67			
8.57				7.83				1.97				1.13			
3.73				7.55				8.25				8.25			
3.53				7.57				9.13				3.39			
4.07				7.65				2.83				8.57			
3.05				3.71				3.53				3.89			
3.75				3.69				7.15				7.11			
7.15				7.63				8.83				3.95			
8.15				7.13				1.29				3.49			
3.27				7.67				8.55				3.37			
7.17								3.07				1.29			
7.19															
3.07															
8.13															

Represented below are the initial features selected by PLS-DA (*VIP *cut-off ≥ 1) and Random Forest (RF) feature selection techniques, denoted by FS for classes Day 1 vs. Day 3 and Day 3 vs. Day 5 in Dataset 1. Also represented in bold are the additional features discovered by MetaFIND, denoted by MF. The asterisk signifies those additional features discovered by MetaFIND that were identified as being representative of all or part of a class discriminating metabolite. The original VIP scores/RF Importance Rank (Imp. Rank) of the features discovered by MetaFIND are also given.

### Dataset 2: brain region study

Feature selection was carried out on Dataset 2 for each brain region class comparison. Dataset 2 contains many more features than Dataset 1 and as a result a greater number of significant features are retrieved by PLS-DA and Random Forest feature selection methods. PLS-DA selected 41 features that discriminated between the brain stem and cerebellum classes, 32 features for the brain stem and hippocampus classes and 28 for the hippocampus and cerebellum classes. Random Forest feature selection retrieved 100 discriminating features for each class comparison.

**Figure 7:**
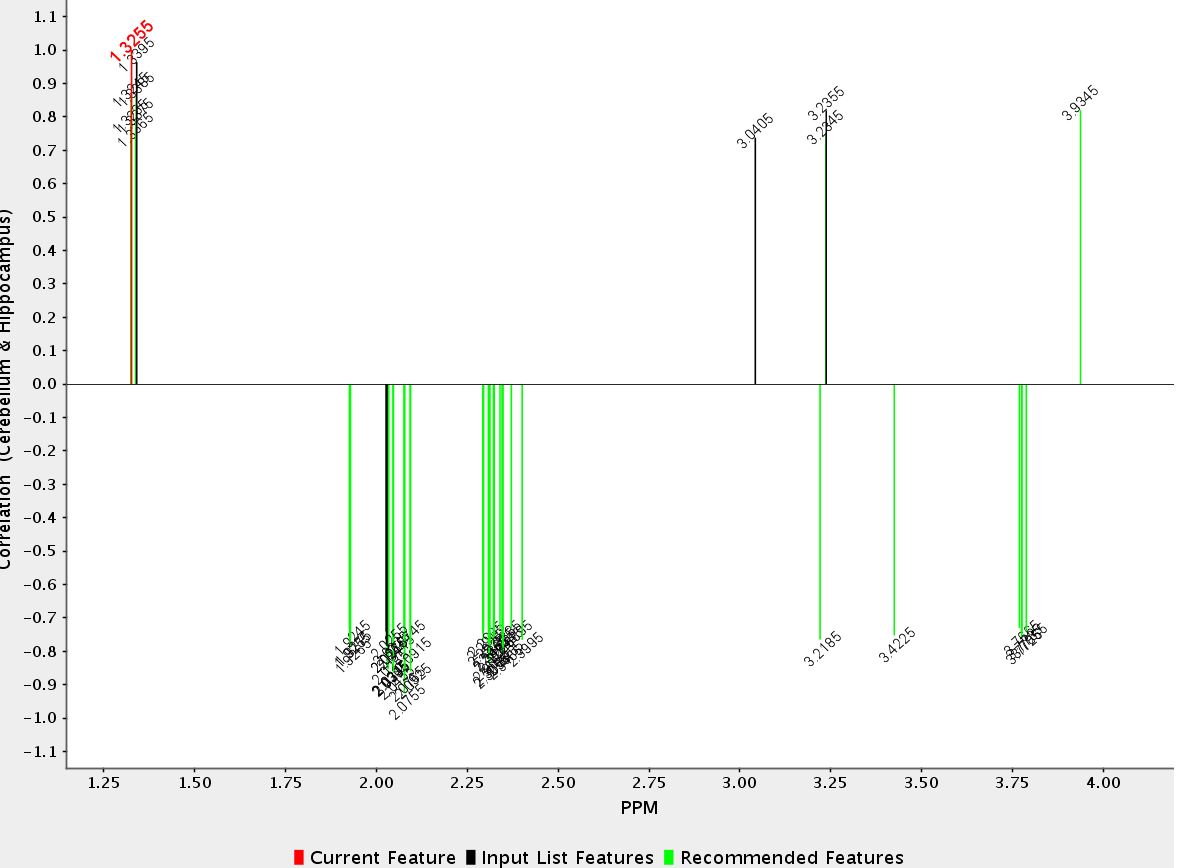
**Analysis of Dataset 2 using MetaFIND**. Visualization of the features that were highly correlated (*r *= +/- 0.73) to reference feature 1.326 (red) which represents part of the lactate signature. At this correlation the full lactate signature can be seen. The peaks representing myo-inositol (3.236 ppm), creatinine signature (3.041 ppm & 3.936 ppm) appear above the *x*-axis (positively correlated). Peaks representing NAG (2.060 ppm) and Gln/Glu (2.338 ppm & 2.757 ppm) appear below the *x*-axis (negatively correlated).

MetaFIND was then used to carry out post-feature selection analysis. Figure 7 shows the correlation graph after the features selected by PLS-DA were imported. The cerebellum and hippocampus class comparison was first examined. The reference feature is 1.326 (red) represents part of the lactate signature (1.326 ppm & 1.340 ppm). Lactate is present at higher levels in the cerebellum compared to the hippocampus. As the correlation threshold is lowered the rest of the lactate signature appears first. This is followed by the myo-inositol (3.236 ppm) signature and creatinine signature (3.041 ppm & 3.936 ppm) above the *x*-axis (positively correlated) and the NAG (2.060 ppm) signature and glutamine/glutamate (Gln/Glu) signature (2.338 ppm & 2.757 ppm) below the *x*-axis (negatively correlated). Additional discriminating features (11) were recommended by MetaFIND for this class comparison and can be seen in Table 2. All features represent peaks and separate the classes fully (*p*-value of 7.78E-4.). Additional recommended features, recommended after Random Forest feature selection may also be seen in Table 2. Those marked with an asterisk represent verified peaks missed by Random Forest feature selection but caught by MetaFIND post-feature selection analysis.

**Table 2:** MetaFIND uncovers additional features from Dataset 2 for the Cerebellum vs. Hippocampus class comparison.

**Brain Stem vs. Hippocampus**					
**PLS-DA**			**Random Forest**		
**MetaFIND**	**P-Value**	**VIP**	**MetaFIND**	**P-Value**	**Imp.Rank**
	
2.069	7.78E-04	0.82	1.909	7.78E-04	336
2.192	7.78E-04	0.57	2.061*	7.78E-04	174
2.208	7.78E-04	0.69	2.085	7.78E-04	396
2.223	7.78E-04	0.36	2.100	7.78E-04	368
2.732	7.78E-04	0.65	8.573	7.78E-04	408
3.120	7.78E-04	1.01	1.923	1.92E-03	1077
3.873	7.78E-04	0.09	6.154	8.65E-03	1284
3.987	7.78E-04	1.98	3.236*	1.17E-02	1263
3.994	7.78E-04	2.06	1.326*	4.06E-02	1715
6.161	7.78E-04	0.17	1.341*	4.62E-02	2529
8.246	7.78E-04	0.67	1.340*	5.87E-02	1385
			1.327*	2.94E-01	2521

Additional class discriminating features recommended by MetaFIND after initial PLS-DA (*VIP *cut-off ≥ 5) and Random Forest (RF) feature selection for the Cerebellum vs. Hippocampus class comparison (which returned 28 and 100 features respectively). The MetaFIND column shows class discriminating features that were retrieved using the MetaFIND application. An asterisk signifies that the feature represents part of a metabolite with a known assignment. The original VIP scores/RF Importance Rank (Imp. Rank) of the features discovered by MetaFIND are also given.

**Figure 8:**
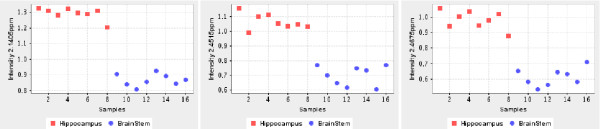
**Features discovered by MetaFIND from Dataset 2**. Feature plots for part of the Glu/Gln signature that was retrieved by MetaFIND analysis of the PLS-DA features in selected for the Brain Stem vs Hippocampus class comparison.

In Table 3, brain stem vs. cerebellum comparison, 19 additional features were recommended by MetaFIND after importing features selected by PLS-DA. Most features separated the classes fully (*p*-value of 7.78E-4.). Of these 1.894 ppm, 1.909 ppm, 1.938 ppm peaks belong to GABA. Additional features (26) were also uncovered by MetaFIND upon importing those retrieved by Random Forest feature selection. These included features which represented the lactate, NAA and taurine metabolites (marked with an asterisk). Similar results can be seen in Table 4, with MetaFIND recommending additional discriminating peaks for brain stem vs. hippocampus comparison, for both PLS-DA and RF feature selection methods. In Table 4 MetaFIND aids retrieval of part of the Glu/Gln peak signature which was omitted by the initial feature selection. The feature plot showing the class separation of these peaks is illustrated in Figure 8.

**Table 3:** MetaFIND uncovers additional features from Dataset 2 for the Brain Stem vs. Cerebellum class comparison.

**Brain Stem vs. Hippocampus**					
**PLS-DA**			**Random Forest**		
**MetaFIND**	**P-Value**	**VIP**	**MetaFIND**	**P-Value**	**Imp.Rank**
	
2.069	7.78E-04	0.75	1.338*	7.78E-04	365
2.085	7.78E-04	0.72	2.039	7.78E-04	156
2.207	7.78E-04	1.09	2.069	7.78E-04	786
2.540	7.78E-04	0.10	2.085	7.78E-04	330
2.765	7.78E-04	0.39	2.540	7.78E-04	117
2.794	7.78E-04	0.52	3.236*	7.78E-04	199
3.908	7.78E-04	0.50	3.237*	7.78E-04	393
6.079	7.78E-04	0.15	3.307	7.78E-04	1458
6.091	7.78E-04	0.15	3.770*	7.78E-04	105
6.161	7.78E-04	0.13	3.771*	7.78E-04	767
8.425	1.63E-03	0.02	3.935*	7.78E-04	398
2.801	3.28E-03	0.54	6.079	7.78E-04	112
3.686	3.28E-03	1.06	3.235*	1.13E-03	684
6.113	3.28E-03	0.34	3.934*	1.63E-03	896
8.247	4.57E-03	1.00	8.425	1.63E-03	734
1.894*	6.32E-03	0.17	3.681	3.28E-03	854
1.909*	1.17E-02	0.64	6.113	3.28E-03	698
1.938*	1.17E-02	0.12	6.161	3.28E-03	364
			3.933*	6.32E-03	1468
			3.289*	8.65E-03	1203
			1.339*	4.57E-02	1589

Additional class discriminating features retrieved using MetaFIND after initial PLS-DA, VIP cut-off ≥ 6, and Random Forest (RF) feature selection for the Brain Stem vs. Cerebellum class comparison (which returned 41 and 100 features respectively). The MetaFIND column shows class discriminating features that were retrieved using the MetaFIND application. An asterisk signifies that the feature represents part of a metabolite with a known assignment. The original VIP scores/RF Importance Rank (Imp. Rank) of the features discovered by MetaFIND are also given.

**Table 4:** MetaFIND uncovers additional features from Dataset 2 for the Brain Stem vs. Hippocampus class comparison.

**Brain Stem vs. Hippocampus**					
**PLS-DA**			**Random Forest**		
**MetaFIND**	**P-Value**	**VIP**	**MetaFIND**	**P-Value**	**Imp.Rank**
	
2.039	7.78E-04	1.52	1.325*	7.78E-04	240
2.069	7.78E-04	2.17	1.339*	7.78E-04	180
2.086	7.78E-04	1.89	1.341*	7.78E-04	1689
2.118	7.78E-04	2.65	1.480	7.78E-04	1033
2.141*	7.78E-04	3.51	1.494	7.78E-04	1659
2.190	7.78E-04	1.78	2.060*	7.78E-04	404
2.207	7.78E-04	2.69	3.218	7.78E-04	591
2.450*	7.78E-04	3.07	3.275*	7.78E-04	933
2.467*	7.78E-04	2.92	3.276*	7.78E-04	109
2.559	7.78E-04	1.40	3.277*	7.78E-04	381
2.732	7.78E-04	1.68	3.278*	7.78E-04	102
2.755	7.78E-04	1.42	3.289*	7.78E-04	1632
3.630	7.78E-04	0.89	3.796	7.78E-04	630
3.646	7.78E-04	3.89	5.940	7.78E-04	482
3.682	7.78E-04	3.50	5.952	7.78E-04	111
3.855	7.78E-04	1.09	6.045	7.78E-04	292
3.873	7.78E-04	0.43	6.079	7.78E-04	214
3.892	7.78E-04	1.18	8.268	7.78E-04	1031
3.994	7.78E-04	2.44	6.034	1.13E-03	1083
6.091	7.78E-04	0.76	6.100	3.28E-03	1274
			6.113	4.57E-03	1168
			1.340*	1.17E-02	1299
			3.307	1.17E-02	1852
			8.247	1.17E-02	1103
			1.326*	2.09E-02	1721
			1.327*	5.87E-02	2480

Additional class discriminating features recommended by MetaFIND after initial PLS-DA, VIP cut-off ≥ 5, and Random Forest (RF) feature selection for the Brain Stem vs. Hippocampus class comparison (which returned 32 and 100 features respectively). The MetaFIND column shows class discriminating features that were retrieved using the MetaFIND application. An asterisk signifies that the feature represents part of a metabolite with a known assignment. The original VIP scores/RF Importance Rank (Imp. Rank) of the features discovered by MetaFIND are also given.

## Conclusion

The high dimensionality and multi-collinear nature of metabolomics data provides a significant challenge for both feature selection and subsequent metabolite annotation. These issues combined with other factors such as experimental noise, scaling and threshold selection may lead to the omission of features relevant to class explanation. To address this risk we have developed the MetaFIND application to enable the investigator to carry out an effective 'post-feature selection' correlation analysis. MetaFIND supports the user in metabolite signature identification, feature discovery and may aid inference of metabolic relationships by identifying highly correlated metabolites.

In this study two diverse feature selection methods, namely PLS-DA and Random Forest, were applied to two metabolomics datasets. In all cases the MetaFIND application aided retrieval of additional class discriminating peaks, some of which were subsequently found to represent relevant class discriminating metabolites. Lastly, MetaFIND supports the investigator in the discovery of correlated metabolites, this information may then aid in the construction of networks. This study has illustrated that the performance of data-driven feature selection methods may be augmented by additional user-driven input as supported by the MetaFIND application.

## Competing interests

The authors declare that they have no competing interests.

## Availability and requirements

Project home page: 

Operating systems: Platform independent

Programming language: Java

Software packages: JFreeChart and JCommons

Any restrictions to use by non-academics: Commercial use license can be obtained by contacting the authors

## Authors' contributions

KB carried out the programming and software design and drafted the manuscript. LB provided domain knowledge, data and application testing and helped to draft the manuscript. PC conceived of the study, and participated in its design and coordination and helped to draft the manuscript. All authors read and approved the final manuscript.
